# Three Novel Species with Peptidoglycan Cell Walls form the New Genus *Lacunisphaera* gen. nov. in the Family Opitutaceae of the Verrucomicrobial Subdivision 4

**DOI:** 10.3389/fmicb.2017.00202

**Published:** 2017-02-13

**Authors:** Patrick Rast, Ines Glöckner, Christian Boedeker, Olga Jeske, Sandra Wiegand, Richard Reinhardt, Peter Schumann, Manfred Rohde, Stefan Spring, Frank O. Glöckner, Christian Jogler, Mareike Jogler

**Affiliations:** ^1^Microbial Cell Biology and Genetics, Leibniz-Institut DSMZ-Deutsche Sammlung von Mikroorganismen und Zellkulturen GmbHBraunschweig, Germany; ^2^Institute for Pharmacology, Toxicology and Clinical Pharmacy, University of TechnologyBraunschweig, Germany; ^3^Max Planck Genome Center, Max Planck Institute for Plant Breeding ResearchKöln, Germany; ^4^Department of Central Services, Leibniz-Institut DSMZ-Deutsche Sammlung von Mikroorganismen und Zellkulturen GmbHBraunschweig, Germany; ^5^Central Facility for Microscopy, Helmholtz Centre for Infection ResearchBraunschweig, Germany; ^6^Department Microorganisms, Leibniz-Institut DSMZ-Deutsche Sammlung von Mikroorganismen und Zellkulturen GmbHBraunschweig, Germany; ^7^Department of Molecular Ecology, Max Planck Institute for Marine MicrobiologyBremen, Germany; ^8^Department of Microbiology, Institute for Water and Wetland Research, Faculty of Science, Radboud UniversityNijmegen, Netherlands

**Keywords:** peptidoglycan, subdivision 4, Verrucomicrobia, *Lacunisphaera*, ornithine

## Abstract

The cell wall of free-living bacteria consists of peptidoglycan (PG) and is critical for maintenance of shape as dissolved solutes cause osmotic pressure and challenge cell integrity. Surprisingly, the subdivision 4 of the phylum Verrucomicrobia appears to be exceptional in this respect. Organisms of this subdivision are described to be devoid of muramic or diaminopimelic acid (DAP), usually found as components of PG in bacterial cell walls. Here we describe three novel bacterial strains from a freshwater lake, IG15^T^, IG16b^T^, and IG31^T^, belonging to a new genus in the subdivision 4 of Verrucomicrobia which we found to possess PG as part of their cell walls. Biochemical analysis revealed the presence of DAP not only in these novel strains, but also in *Opitutus terrae* PB90-1^T^, the closest described relative of strains IG15^T^, IG16b^T^, and IG31^T^. Furthermore, we found that nearly all genes necessary for peptidoglycan synthesis are present in genomes of subdivision 4 members, as well as in the complete genome sequence of strain IG16b^T^. In addition, we isolated and visualized PG-sacculi for strain IG16b^T^. Thus, our results challenge the concept of peptidoglycan-less free-living bacteria. Our polyphasic taxonomy approach places the novel strains in a new genus within the family *Opitutaceae*, for which the name *Lacunisphaera* gen. nov. is proposed. Strain designations for IG15^T^, IG16b^T^ and IG31^T^ are *Lacunisphaera parvula* sp. nov. (=DSM 26814 = LMG 29468), *L. limnophila* sp. nov. (=DSM 26815 = LMG 29469) and *L. anatis* sp. nov. (=DSM 103142 = LMG 29578) respectively, with *L. limnophila* IG16b^T^ being the type species of the genus.

## Introduction

In aquatic environments, abiotic factors such as salinity and temperature, but also intrinsic metabolism-related mechanisms challenge the cellular integrity of microorganisms and their ability to proliferate. Protective elements may be of a structural nature, such as S-layers, or the avoidance of osmotic stress by living in dependency of host organisms which provide stable conditions for survival (Miles, 1992; Engelhardt, 2007). Members of the class Mollicutes for example lack a peptidoglycan cell wall (Razin, 2006), are osmotically fragile and exhibit pleomorphism (Miles, 1992). Thus, they depend on an eukaryotic host to provide an osmotically stable environment for living.

On the other hand, free-living bacteria usually possess cell wall structures including three dimensionally cross-linked polymeric glycan strands, interconnected by short peptide elements, a structure commonly known as peptidoglycan (PG) to protect cellular integrity. Among bacteria only few exceptions are described while all controversy discussed species belong to the Planctomycetes-Verrucomicrobia-Chlamydiae (PVC) superphylum (Wagner and Horn, 2006). In many respects, this PVC-superphylum seems to challenge our concept of the prokaryotic cell (Lee et al., 2009; Fuerst and Sagulenko, 2011; Jacquier et al., 2015; Rivas-Marín et al., 2016). In particular, the suggested absence of PG in Planctomycetes (König et al., 1984), Chlamydia (Fox et al., 1990) and subdivision 4 Verrucomicrobia (Yoon, 2011) is remarkable. While the assumed lack of PG seems to be associated with the lack of the otherwise universal bacterial cell division protein FtsZ in Planctomycetes (Pilhofer et al., 2008; Jogler et al., 2012) and Chlamydia (Stephens et al., 1998), subdivision 4 Verrucomicrobia encode the tubulin homolog FtsZ (Pilhofer et al., 2008). However, Planctomycetes were recently found to possess a PG cell wall (Jeske et al., 2015; van Teeseling et al., 2015). For Chlamydia, the existence of PG was demonstrated but a canonical PG sacculus was not isolated (Liechti et al., 2014; Packiam et al., 2015). However, for some other members of the phylum Chlamydiae a PG sacculus was identified (Pilhofer et al., 2013). Chlamydia are obligate intracellular pathogens (Jacquier et al., 2015) and thus dwell in an environment isotonic to their cytoplasm, they do not necessarily require a peptidoglycan sacculus to maintain cell shape. Accordingly, recent evidence suggests that PG forms an MreB regulated ring at mid-cell to allow cell division in pathogenic Chlamydia (Liechti et al., 2016). In contrast a typical bacterial sacculus was reported for the free-living Planctomycetes that have to withstand various osmotic challenges in their natural habitats (Jeske et al., 2015; van Teeseling et al., 2015), while free-living bacteria of the verrucomicrobial subdivision 4 are still considered to lack a PG sacculus. This bacterial group belongs to the phylum Verrucomicrobia which is divided into six so-called subdivisions. Thus far, cultured representatives are available for subdivisions 1–4. Recently for subdivision 5 the new Phylum Kiritimatiellaeota was proposed, with one characterized isolate (Spring et al., 2016). Playing a crucial role in environmental nutrient cycles, members of the Verrucomicrobia have not only been found to degrade a variety of complex polymeric compounds in, e.g., soil communities (Wang et al., 2014, 2015), some were also identified as methanotrophs (Sharp et al., 2013; van Teeseling et al., 2014). Increasing efforts to extend the knowledge about this environmentally important phylum have led to the successful isolation and description of several new species in recent years (Lee et al., 2014; Kim et al., 2015). However, the majority of new strains brought into pure culture is affiliated with subdivision 1. Therefore, the scarce data existing to date leaves inconclusive results about the suspected peptidoglycan anomaly of subdivision 4 Verrucomicrobia. Furthermore, thus far only two genomes from validly described species (*Opitutus terrae* and *Coraliomargarita akajimensis*) are available. Both genomes were not yet analyzed for PG related genes with state-of-the-art bioinformatic methods (Jeske et al., 2015). Some members of this subdivision have been found to be resistant to various β-lactam antibiotics, indicating either absence of PG or an resistance mechanism such as β-lactamases. For other strains the presence of typical cellular PG building blocks was not investigated at the time of their description (Shieh and Jean, 1998; Choo et al., 2007), leaving open the question whether peptidoglycan exists in verrucomicrobial subdivision 4. Members of this subdivision have been isolated from soil communities and leafs, while most strains originate from aquatic habitats, including freshwater lakes, marine waters and extreme habitats such as hot springs (Shieh and Jean, 1998; Chin et al., 2001; Choo et al., 2007; Yoon et al., 2007c, 2010). Here we describe the targeted isolation of subdivision 4 Verrucomicrobia, using antibiotic agents as selective markers for β-lactam resistant bacteria. Our strategy led to the successful cultivation of three novel strains from surface fresh water samples. By biochemical, microscopic and computational analysis we found that the novel and previously reported members of the verrucomicrobial subdivision 4 possess PG as part of their cell walls.

Our findings challenge the proposed absence of peptidoglycan among subdivision 4 Verrucomicrobia, while at the same time extending the scarce pool of cultivated species in this environmentally important phylum.

## Materials and Methods

### Sample Collection and Preparation

Surface freshwater samples were collected in triplicates from a local pond (52° 9′ 38″ N, 10° 32′ 40″ E, Wolfenbüttel, Germany) on August 30th, 2012 after the observation of a massive cyanobacterial blooming event. Water was collected in sterile polypropylene bottles, immediately transferred to the laboratory, homogenized and processed within 2 h.

### Culture Media and Bacterial Isolation

Cultivation medium M1H was prepared with double distilled water containing 0.25 g/l peptone (Bacto^TM^), 0.25 g/l yeast extract (Bacto^TM^), 2.38 g/l HEPES (Serva), 20 ml/l mineral salt solution and a pH adjusted to 8.0 with 5 M KOH. After sterilization, the medium was complemented with 10 ml/l of a 2.5% glucose solution, 5 ml/l double concentrated vitamin solution, 1 ml/l of 100 mg/ml carbenicillin and 20 mg/ml cycloheximide stock solutions, respectively. Solid medium was prepared with three times washed 12 g/l agar (Bacto^TM^) and cooled to 55°C prior to the addition of heat sensitive solutions. Both, mineral salt solution and double concentrated vitamin solution were prepared according to DSMZ medium 621, while metal salts solution consisted of 250 mg/l Na–EDTA, 1095 mg/l ZnSO_4_^.^7H_2_O, 500 mg/l FeSO_4_^.^7H_2_O, 154 mg/l MnSO_4_^.^H_2_O, 39.5 mg/l CuSO_4_^.^7H_2_O, 20.3 mg/l CoCl_2_^.^6H_2_O, and 17.7 mg/l Na_2_B_4_O_7_^.^10H_2_O of which 50 ml were added per liter of mineral salt solution.

For initial bacterial isolation, solid M1H medium was supplemented with 100 μl of carbenicillin stock solution (100 mg/ml), dried for 30 min and inoculated with 100 μl homogenized sample material per plate in a 10–10^-2^ dilution series and incubated at 20°C in the dark until colony formation became visible. Single colonies were inoculated on fresh solid medium with respective antibiotics. Pure cultures were cryopreserved in M1H medium supplemented with 50% glycerol or 5% DMSO and stored at -80°C. Strains isolated and later identified as members of the verrucomicrobial subdivision 4 were designated IG15^T^, IG16b^T^ and IG31^T^. Unless otherwise indicated, verrucomicrobial strains were cultivated at 28°C to ensure reproducibility of cultivation dependent experiments.

Cultivation medium of thin layer chromatography (TLC) reference strains, *Bacillus subtilis* DSM 10 and *Escherichia coli* DSM 498, was standard LB medium contained 10 g/l tryptone, 10g/l sodium chloride and 5 g/l yeast extract at pH 7.0 (Bertani, 1951).

For *O. terrae* PB90-1^T^ cultivation was performed following the recommendations of the Leibniz Institute DSMZ (DSMZ medium no. 295).

### Molecular Identification and Phylogenetic Analysis

Novel isolates were identified by direct sequencing of the 16S rRNA gene after amplification with the optimized universal primers 8f (5′–AGA GTT TGA TCM TGG CTC AG–3′) and 1492r (5′–GGY TAC CTT GTT ACG ACT T–3′) modified from (Lane, 1991). PCR reactions were performed directly on single colonies for identification or liquid cultures to check for purity, using the *Taq* DNA Polymerase Kit (Qiagen) with one reaction of 25 μl containing 11 μl PCR–grade H_2_O, 2.5 μl 10x CoralLoad buffer, 2.5 μl Q-Solution, 0.5 μl dNTPs (10 mM each), 1 μl sterile bovine serum albumin solution (20 mg/ml), 0.5 μl MgCl_2_ solution (25 mM), 0.125 μl *Taq*–Polymerase (1 U/μl) and 1 μl of each primer (10 pmol). The employed protocol consisted of two steps, the first step with an initial denaturation at 94°C, 5 min, 10 cycles of denaturation at 94°C, 30 s, annealing at 59°C, 30 s, elongation at 72°C, 1 min, followed by the second step with 20 cycles denaturation at 94°C, 30 s, annealing at 54°C, 30 s, elongation at 72°C, 1 min and a final elongation step at 72°C, 7 min. All PCRs were carried out in an Applied Biosystems^®^ Veriti^®^ thermal cycler (Thermo Fisher Scientific) and PCR products were stored at 4°C until Sanger sequencing.

To generate near full length 16S sequences, additional primers (compare **Supplementary Table S1**) were used for sequencing and assembly of the resulting sequences was performed with the ContigExpress application of the Vector NTI^®^ Advance 10 software (Thermo Fisher Scientific).

Alignment of near full length 16S rRNA sequences was performed using the SINA web aligner (Pruesse et al., 2012), corrected manually and used for phylogenetic tree reconstruction. Tree reconstruction was performed with the ARB software package (Ludwig et al., 2004) using the Maximum Likelihood RAxML module and rate distribution model GTR GAMMA running the rapid bootstrap analysis algorithm, the Neighbor Joining tool with Felsenstein correction for DNA and Maximum Parsimony method employing the Phylip DNAPARS module. Bootstrap values for all three methods were computed with 1,000 resamplings including the *E. coli* 16S rRNA gene positions 101–1,371. The analysis involved 68 nucleotide sequences of described type strains and uncultured clones, related to the novel strains (compare **Supplementary Table S2**). 16S rRNA gene identity values of novel isolates and related type strains were calculated using neighbor joining clustering of the ARB package.

### Characterization of Novel Isolates

#### Morphological, Physiological, and Biochemical Analysis

Bacterial cells were immobilized on a 1% agarose–pad in MatTek 35 mm glass-bottom dishes and imaged under phase–contrast illumination using a Nikon Eclipse Ti invers microscope at 100× magnification and the Nikon DS–Ri2 camera. To determine the cell size of the novel strains, 100 individual cells of each strain were measured using the NIS-Elements software V4.3 (Nikon Instruments).

For field emission scanning electron microscopy (FESEM) bacteria were fixed in 1% formaldehyde in HEPES buffer (3 mM HEPES, 0.3 mM CaCl_2_, 0.3 mM MgCl_2_, 2.7 mM sucrose, pH 6.9) for 1 h on ice and washed one time with HEPES buffer. Cover slips with a diameter of 12 mm were coated with a poly-L-lysine solution (Sigma–Aldrich) for 10 min, washed in distilled water and air-dried. 50 μl of the fixed bacteria solution was placed on a cover slip and allowed to settle for 10 min. Cover slips were then fixed in 1% glutaraldehyde in TE buffer (20 mM TRIS, 1 mM EDTA, pH 6,9) for 5 min at room temperature and subsequently washed twice with TE–buffer before dehydrating in a graded series of acetone (10, 30, 50, 70, 90, and 100%) on ice for 10 min at each concentration. Samples from the 100% acetone step were brought to room temperature before placing them in fresh 100% acetone. Samples were then subjected to critical-point drying with liquid CO_2_ (CPD 300, Leica). Dried samples were covered with a gold/palladium (80/20) film by sputter coating (SCD 500, Bal–Tec) before examination in a field emission scanning electron microscope (Zeiss Merlin) using the Everhart Thornley HESE2–detector and the inlens SE–detector in a 25:75 ratio at an acceleration voltage of 5 kV.

Temperature optima of the novel isolates were determined by optical density measurements of growing cultures at 600 nm (OD_600nm_). Strains were inoculated 1:10 from early stationary phase cultures in glass tubes with M1H medium and incubated under constant agitation in temperature controlled shakers (for exact temperatures tested, compare **Supplementary Figure S1**). Measurements were performed in triplicates and each tube served as its own blank prior to inoculation. Resulting growth curves were analyzed by plotting change of OD_600nm_ during exponential growth phases (slope values), of each individual temperature against temperature values in °C.

To determine the pH optimum, M1H medium was buffered to pH values of 5.0, 6.0, 6.5, 7.0, 7.5, 8.0, 8.5, 9.0, and 10.0 using 10 mM MES, HEPES, HEPPS and CHES buffers, corresponding to their individual buffer range. OD_600nm_ was determined in glass tubes, incubated at 28°C, with three replicates as measure of growth. Catalase activity was determined by bubble formation with fresh 3% H_2_O_2_ solution. Cytochrom oxidase activity was determined using Bactident^®^ Oxidase test stripes (Merck Millipore) following the manufacturer’s instructions. Gram properties were determined by reaction of fresh biomass with fresh 3% KOH solution (Suslow et al., 1982).

Substrate utilization of the isolated strains was investigated using the Biolog GN2 MicroLog^TM^ test panel for Gram-negative bacteria. Sterile glass tubes were prepared in duplicates with a basic medium mixture containing 15.7 ml IF-0a inoculation fluid (Biolog), 160 μl of 1 M HEPES buffer (pH 8.0) and 80 μl double concentrated vitamin solution. Tubes were inoculated with bacterial colony material from exponentially growing cultures to a turbidity of 56–68%. Two individual plates per strain were evaluated. To enable the comparison of the derived data, the data of each single experiment were normalized to 100. Only values corresponding to >25% utilization were considered as positive. The heat map graphic was obtained in the R environment (R Core Team, 2015) by using the heatmap.2() function of the gplots package.

#### Analysis of Cellular Fatty Acids

Biomass of the isolated strains was obtained from liquid cultures grown in M1H medium at 28°C until stationary phase. The obtained biomasses were stored at -20°C. For fatty acid analysis, 30 mg of lyophilized biomass was processed according to the standards of the Identification Service of the German Collection of Microorganisms and Cell Cultures (DSMZ) (Miller, 1982; Kuykendall et al., 1988).

#### Determination of Molar G + C Content

Strains were grown in liquid culture to stationary phase and biomass was obtained by centrifugation. For strains IG15^T^ and IG31^T^, the molar G + C content was determined by the service facilities of the DSMZ. In brief, genomic DNA is isolated (Cashion et al., 1977), hydrolyzed, dephosphorylized (Mesbah et al., 1989) and analyzed by HPLC (Tamaoka and Komagata, 1984) in comparison to DNA standards from organisms with published genome sequences and a G + C content range from 43 to 72 mol%. G + C content of strain IG16b^T^ was determined during genome sequencing with the Pacific Bioscience sequencer.

#### Antibiotic Susceptibility

Tolerance of IG15^T^ and IG16b^T^ toward β-lactam antibiotic agents was investigated in a treatment assay using carbenicillin. Strains were inoculated as triplicates 1:10 in glass tubes with M1H medium and final concentrations of 0, 500, 1000, or 2000 mg/l carbenicillin were added. Tubes were incubated at 28°C and growth was measured as change in optical density at 600 nm. After 120 h of incubation, cell viability was investigated by FESEM and cell numbers per ml were calculated by counting with a Neubauer chamber.

### Genome Sequencing of Strain IG16b^T^

#### DNA Extraction and Purity Control

To obtain high molecular weight DNA of strain IG16b^T^, nucleic acid was extracted from whole-cells using a tweaked Genomic DNA kit protocol with Genomic tips 100/G (Qiagen). The protocol was performed as recommended by the manufacturer with one exception: incubation time with digestive enzymes was prolonged to an overnight step to ensure complete lysis of bacterial cells. An aliquot of the extracted DNA was used to prepare 16S rRNA clone libraries (Zero Blunt^®^ PCR Cloning kit; Invitrogen) and resulting clones were sequenced to ensure purity of the extracted DNA.

#### Sequencing and Gene Content Analysis

*De novo* genome sequencing of strain IG16b^T^ was performed using a PacBio RS sequencer. Single molecule real-time (SMRT) bell^TM^ libraries (Pacific Bioscience) were prepared using ∼10 μg genomic DNA. Sequencing data was processed and assembled using the SMRT analysis software. The closed and complete chromosome of strain IG16b^T^ was annotated using the Prokka annotation tool (Seemann, 2014) and subjected to analysis for putative genomic islands and phage regions using IslandViewer3 (Dhillon et al., 2015) and PHAST (Zhou et al., 2011), respectively. The verrucomicrobial genomes for the gene content analysis were derived from NCBI and IMG (Markowitz et al., 2012) in April 2016 and had to match the following criteria upon CheckM analysis (Parks et al., 2015): completeness > 90, contamination < 5 and strain heterogeneity < 20. Orthologs were detected by Proteinortho (Lechner et al., 2011), a tool that identifies the reciprocal best hits from the given protein sequences. The genome plot was then generated with BRIG (Alikhan et al., 2011).

### Peptidoglycan Analysis

#### Identification of Peptidopglycan Synthesis Genes and β-lactamase Protein Homologs

The presence of peptidoglycan synthesis genes was analyzed using blastp (Altschul et al., 1997), while protein sequences of *Phycisphaera mikurensis* FYK2301M01^T^ or *Gimesia maris* 534-30^T^ served as query and were compared with protein sequences encoded in the genomes of *O. terrae* PB90-1^T^, *C. akajimensis* 04OKA010-24^T^ and strain IG16b^T^. β-lactamase encoding genes were detected in IG16, *O. terrae* and *C. akajimensis* as previosly described (Bush, 2013; Jeske et al., 2015). For both analysis, homologous proteins required an identity >30%, an *e*-value lower than 1e^-6^ and a conserved domain architecture.

#### Lysozyme Assay

Susceptibility to lysozyme was investigated by incubation of the novel strains in M1H medium. Since strain IG31^T^ showed no lysis after 24 h in M1H medium, osmotic stress was increased by incubation of cells in ddH_2_O (negative controls as well as lysozyme treated cells). Lysozyme was added to a final concentration of 10 mg/ml and cells were incubated for up to 24 h at 37°C under constant agitation at 300 rpm. Bacterial cells were immobilized on a 1% agarose–pad in MatTek 35 mm glass–bottom dishes and imaged under phase–contrast illumination using a Nikon Eclipse Ti invers microscope at 100× magnification and the Nikon DS–Ri2 camera (Nikon Instruments). Cell viability was checked by microscopy after 1, 3, 6, and 24 h of incubation in M1H medium or ddH_2_O until cell lysis was observed.

#### Biochemical Analysis of Peptidoglycan Building Blocks

The presence of diaminopimelic acid (DAP) was investigated employing thin-layer chromatography and gas chromatography/mass spectrometry (GC/MS). Thin-layer chromatography of whole-cell hydrolysates of strains IG15^T^, IG16b^T^, IG31^T^ as well as reference strains *B. subtilis* DSM 10 and *E. coli* DSM 498 was performed as previously described (Staneck and Roberts, 1974). Novel isolates were grown in M1H medium at 28°C to stationary phase and cells were harvested by centrifugation. *B. subtilis* and *E. coli* served as organismic controls, grown in 50 ml LB medium at 37°C overnight and harvested by centrifugation, while a mixture of purified DAP isomers (Sigma) was used as detection standard.

Whole-cell hydrolysates of strains IG15^T^, IG16b^T^, IG31^T^ as well as of *O. terrae* PB90-1^T^ (DSM 11246) were analyzed using a gas chromatography/mass spectrometry (GS/MS)-based method (Schumann, 2011), previously employed to quantify the peptidoglycan marker DAP and in addition ornithine in a new proposed Verrucomicrobia related phylum (Spring et al., 2016). In brief, cell pellets were obtained from liquid cultures (grown as described above) and biomass was lyophilized. Samples were standardized for the quantification of diagnostic diamino acids by supplementing lyophilized biomass with 2 μmol of norleucine as internal standard. The hydrolysates (200 μl 4N HCl, 100°C, 16 h) of the samples were dried in a vacuum desiccator. Amino acids derivatized to N-heptafluorobutyryl isobutylesters and were resolved in ethyl acetate and analyzed by GC/MS (Singlequad 320, Varian; electron impact ionization, scan range 60 to 800 m/z). The DAP derivative was detected in extracted ion chromatograms using the characteristic fragment ion set 380, 324, 306, and 278 m/z at a retention time of 22.17 min. A fragment ion of 266 m/z with a retention time of 15.13 min was indicative of the presence of ornithine.

#### Preparation of IG16b^T^ Sacculi

Cells of IG16b^T^ were harvested from 2 l of stationary phase cultures grown in M1H medium at 28°C, by centrifugation at room temperature following a protocol established by van Teeseling et al. (2015). In brief, cells were boiled at 100°C for 1 h with 4% SDS, while being gently mixed by inverting the reaction tube several times in 15 min intervals. Lysates were transferred to Float-a-Lyzer^®^ dialysis tubes (SpectrumLabs, DG Breda, Netherlands) and dialyzed against deionized water in a five-liter beaker over the course of 3 days (water was exchanged two times). Dialyzed samples were stored at RT until analysis by transmission electron microscopy (TEM).

#### Negative Staining of IG16b^T^ Sacculi

Thin carbon support films were prepared by sublimation of a carbon thread onto a freshly cleaved mica surface. Lysate containing the sacculi was adsorbed onto a carbon film for 1 min and negatively stained with 1% (w/v) aqueous uranyl acetate, pH 5.0 (Valentine et al., 1968). After air-drying, samples were examined in a TEM 910 transmission electron microscope (Carl Zeiss, Oberkochen, Germany) at an acceleration voltage of 80 kV and calibrated magnifications using a line replica. Images were recorded digitally with a Slow-Scan CCD-Camera (ProScan, 1024x1024, Scheuring, Germany) with ITEM-Software (Olympus Soft Imaging Solutions, Münster, Germany).

### Nucleotide Sequence Accession Numbers

Near full-length sequences of the 16S ribosomal RNA genes as well as the complete genome sequence of strain IG16b^T^ were deposited with the National Center for Biotechnology Information (NCBI) and are available under KX058881 (IG15^T^), KX058882 (IG16b^T^), KX058883 (IG31^T^) and CP016094 (IG16b^T^ whole genome).

## Results

### Novel Species of the Verrucomicrobial Subdivision 4

#### Isolation and Identification

**FIGURE 1:**
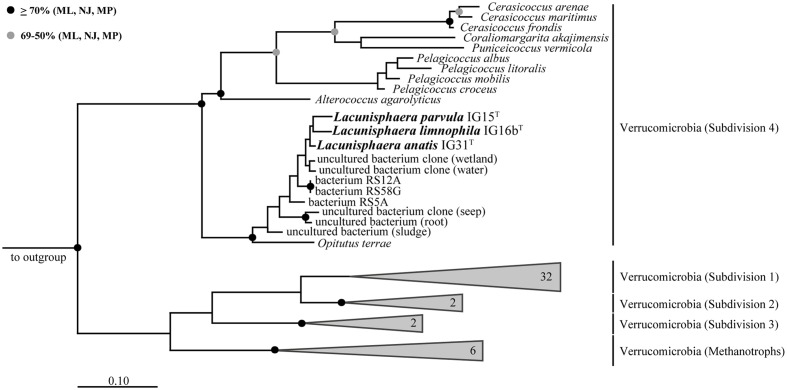
**Maximum likelihood 16S rRNA gene-based phylogenetic tree.** All three strains cluster within the subdivision 4 of Verrucomicrobia with *Opitutus terrae* as the closest representative with validly published name. Related sequences of uncultured bacteria are shown for comparison and pronounce the distinct phylogenetic position of strains IG15^T^, IG16b^T^, and IG31^T^. Bootstrap values based on three different tree building methods (Maximum Likelihood: ML; Neighbor Joining: NJ; Maximum Parsimony: MP). Black dots indicate support values above 70% for all three methods while gray dots show support values of more than 50% for all three methods and less than 70%, at least for one method. Branches that were not supported by all three methods show no dot. Scale bar indicates 10% estimated sequence divergence.

Surface water samples from a local duck pond were used for the targeted isolation of novel subdivision 4 Verrucomicrobia. Given that members of subdivision 4 were thought to lack peptidoglycan, β-lactam antibiotics were used as selection pressure to enrich target bacteria. Obtained colonies of β-lactam resistant bacteria were screened by 16S rRNA gene sequencing analysis and three isolates were identified as members of the verrucomicrobial subdivision 4. Phylogenetic tree reconstruction based on near full-length 16S rRNA gene sequences (**Figure 1**) revealed that strains IG15^T^, IG16b^T^, and IG31^T^ belong to the family of *Opitutaceae*, sharing 92.21, 92.39, and 92.90% sequence identity with the closest related species *O. terrae* PB90-1^T^, respectively (**Table 1**). Based on recent threshold values for 16S rRNA gene sequence comparison (Rosselló-Móra and Amann, 2015), the novel strains represent three distinct species that form a novel genus within the family *Opitutaceae*, with IG15^T^, IG16b^T^, and IG31^T^ being the type strains.

**Table 1:** 16S rRNA gene sequence identity matrix indicating similarity between the three verrucomicrobial isolates and the next relative *Opitutus terrae* PB90-1.

16S rRNA gene sequence similarity [%]	*Opitutus terrae* PB90-1^T^	IG15^T^	IG16b^T^	IG31^T^
*Opitutus terrae* PB90-1^T^	100	92.21	92.39	92.90
IG15^T^	92.21	100	97.07	97.55
IG16b^T^	92.39	97.07	100	97.71
IG31^T^	92.90	97.55	97.71	100


*Similarity values were calculated using neighbor joining clustering.*

#### Morphological, Physiological, and Biochemical Characterization of Novel Strains

**FIGURE 2:**
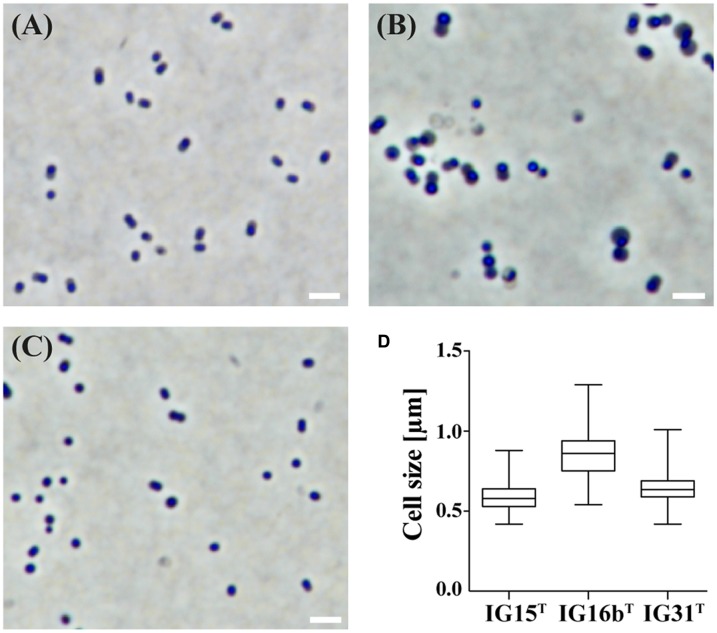
**Investigation of cell morphology and size by light microscopy.** The morphology and average cell size of IG15^T^ **(A)**, IG16b^T^ **(B)**, and IG31^T^ **(C)** was investigated by light microscopy under phase-contrast illumination. Cells of strain IG15^T^ **(A)**, IG16b^T^ **(B)**, and IG31^T^ **(C)** are of coccoid morphology and grow as mono- or diplococci. Cell size was determined by measuring 100 individual cells per strain **(D)** and average cell size with standard deviation differed from 0.6 ± 0.1 μm (IG15^T^), 0.9 ± 0.2 μm (IG16b^T^) to 0.6 ± 0.1 μm (IG31^T^). Scale bar indicates 2 μm.

**FIGURE 3:**
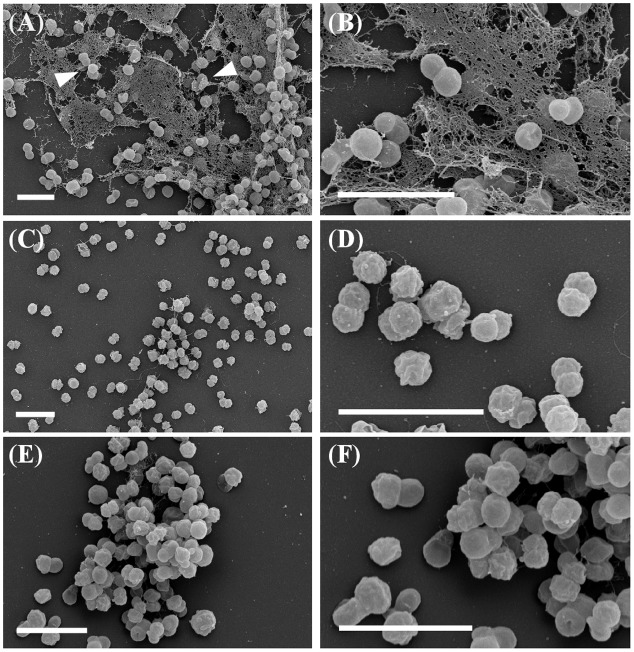
**Field emission scanning electron microscopy.** Strains show coccoid morphology with organization as mono- or diplococci. Micrographs of IG15^T^ cells (**A**, overview; **B**, close up) illustrate the formation of multicellular aggregates embedded in an extracellular matrix substance (white arrowheads). In contrast, cells of IG16b^T^ (**C**, overview; **D**, close up) or IG31^T^ (**E**, overview; **F**, close up) did not produce an extracellular matrix. Scale bar indicates 2 μm.

**FIGURE 4:**
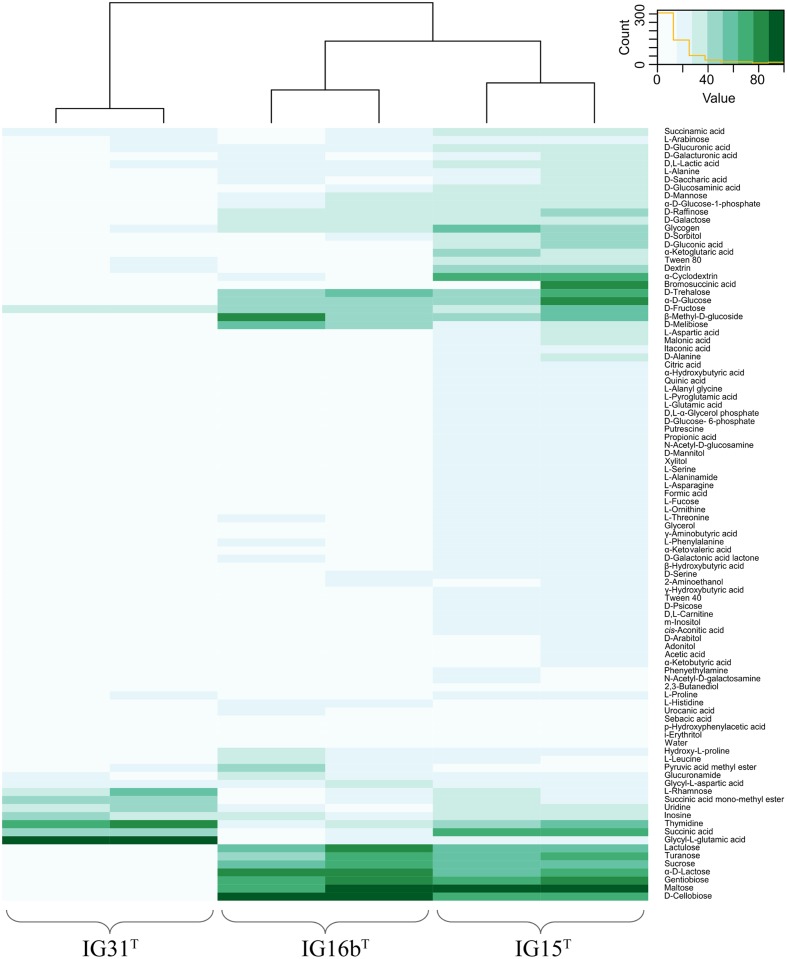
**Heatmap illustration of substrate utilization.** Substrate utilization was tested using the GN2 Microlog^TM^ plate system. Substrate spectrum of IG15^T^ was more similar to IG16b^T^, while some substrates such as succinic acid and α-cyclodextrin were almost solely degraded by IG15^T^. IG16b^T^ in contrast was able to utilize D-cellobiose and α-D-lactose, distinguishing it from strains IG15^T^ and IG31^T^. The utilization pattern of IG31^T^ was less broad, encompassing eight of the 95 tested substrates, but included for example glycyl-L-glutamic acid, which was not utilized by IG15^T^ or IG16b^T^.

Cells of strains IG15^T^, IG16b^T^, and IG31^T^ were investigated using light microscopic and electron microscopic techniques, revealing a coccoid cell shape with cells present as mono- or diplococci (**Figures 2** and **3**). No chain or rosette formation was observed. IG15^T^ cells were the smallest of the three strains in average, measuring 0.6 ± 0.1 μm (diameter of single cocci with standard deviation; *n* = 100 cells) while cells of IG16b^T^ and IG31^T^ measured 0.9 ± 0.2 and 0.6 ± 0.1 μm in diameter, respectively (**Figure 2D**). In wide-field microscopy experiments, cell size variability of all three strains (compare **Figure 2**) became more evident than in scanning electron microscopy, where cells appeared smaller in size (compare **Figure 3**) due to osmotic stress during fixation. During exponential growth, cells of strain IG15^T^ and IG31^T^ were highly motile, while IG16b^T^ showed only very few motile cells. While culture agitation was not necessary for growth, cells of strain IG15^T^ produced an extracellular matrix when grown under constant agitation (90 rpm) (**Figures 3A,B**) with cells embedded in loose aggregates. No extracellular matrix formation was observed for strain IG16b^T^ and IG31^T^ (**Figures 3C–F**). All strains grow aerobically. Temperature and pH optima measurements revealed a mesophilic growth profile with growth temperatures from 13–38, 13–36, and 20–36°C for strains IG15^T^, IG16b^T^, and IG31^T^, respectively. Optical density changes during exponential growth pointed to optimum growth temperatures of 33, 32, and 30°C, respectively (**Supplementary Figure S1**). IG15^T^ and IG16b^T^ were able to grow in pH ranges from 6.0 to 9.0, with an optimum between 7.5 and 8.0. The pH optimum for strain IG31^T^ was not determined, since its pH growth properties are likely to be similar to strains IG15^T^ and IG16b^T^. Additionally, results of the oxidase assays were positive and determination of catalase activity showed negative results for all three strains. Strains were found to be Gram-negative by reaction with 3% KOH solution (Suslow et al., 1982). Substrate utilization profiles of strain IG15^T^ and IG16b^T^ showed similar patterns in terms of sugar and sugar acid utilization, while strain IG31^T^ was clearly distinct, utilizing substrates such as glycyl-L-glutamic acid, L-rhamnose and succinic acid mono-methyl ester (**Figure 4**). Cellular fatty acid analysis identified iso-C_15:0_ as major component of IG15^T^ and IG16b^T^ cell walls with 33.3 and 48.6%, respectively, while IG31^T^ only contained 9.1% of this particular fatty acid (**Supplementary Table S3**). Furthermore, IG31^T^ possessed iso-C_14:0_ as major component (15.4%).

#### Antibiotic Susceptibility of Strains IG15^T^ and IG16b^T^

**FIGURE 5:**
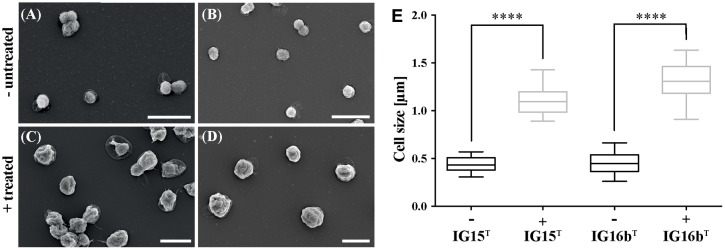
**Effect of carbenicillin treatment on IG15^T^ and IG16b^T^ cells.** Cells of IG15^T^ and IG16b^T^ were treated with carbenicillin for 120 h at 28°C. Untreated cells IG15^T^ and IG16b^T^ **(A,B)** showed no change in morphology, while size of treated cells (**C,D**: 2 mg/ml carbenicillin) was significantly increased **(E)**. Cell size was calculated by counting 20–32 cells per treatment condition. ^∗∗∗∗^ = *p* < 0.0001. Scale bar indicates 2 μm.

Antibiotic susceptibility of strains IG15^T^ and IG16b^T^ toward β-lactams was investigated by treatment with carbenicillin. Optical density (OD_600nm_) measurements indicated growth at all tested antibiotic concentrations for both strains, as values increased over time (**Supplementary Figures S2A,B**). However, size measurements based on SEM micrographs revealed that treated cells of both strains were significantly increased in size when compared to untreated samples (**Figure 5**; *p* = 0.0001). Furthermore, the number of cells per ml was significantly lower (about 10-fold) in treated samples (**Supplementary Figure S2C**; *p* = 0.001). Thus, the increase of OD_600nm_ was rather caused by swelling of the cells, than by multiplication after cell division.

#### Genome Sequencing and Gene Content Analysis of IG16b^T^

**FIGURE 6:**
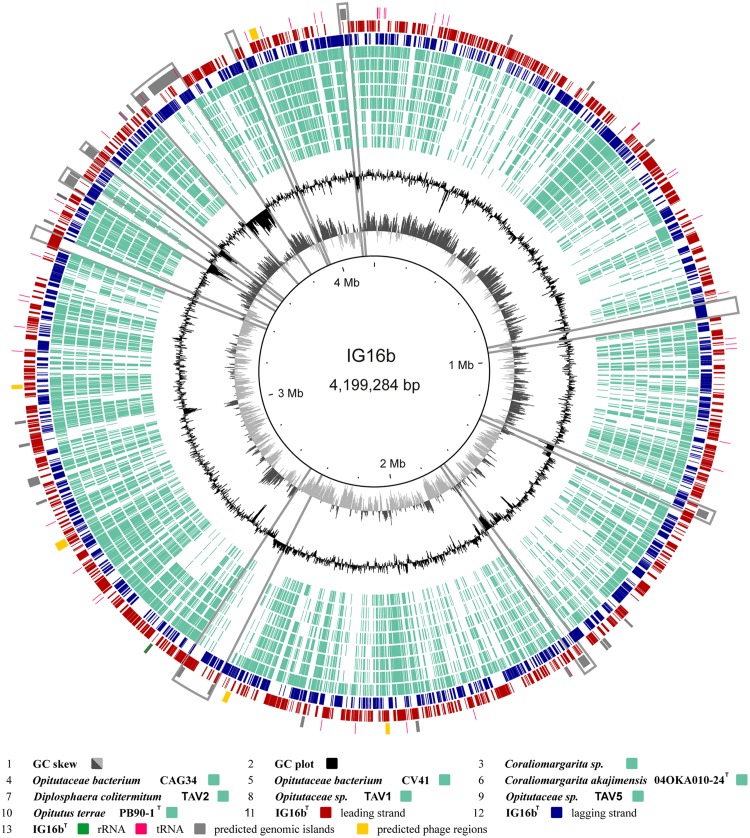
**Circular plot of strain IG16b^T^’s 4,199,284 bp chromosome.** Outer circles display protein (red and blue), tRNA (pink) and rRNA (green) encoding genes as well as predicted genomic islands (gray) and prophage regions (yellow). The inner circles show the GC plot (black) and the GC skew (dark and light gray). Ortholog genes from available genomes of subdivision 4 verrucomicrobial strains, were identified by reciprocal BLAST and are depicted in light turquoise. Notable islands of unique gene content in IG16b^T^ are marked by gray boxes. These regions are often accompanied by a distinct change in GC composition. They mainly hold hypothetical proteins and proteins of unknown function. All predicted prophage regions are incomplete.

The genome of strain IG16b^T^ was obtained solely with single molecule real-time sequencing (PacBio). Sequencing read length was 3823 bp in average and yielded 616 mega bp of sequencing data from 6 SMRT cells with a coverage of ∼80× per base. Chromosome size was determined at 4,199,284 bp in length and bear a GC content of 66.5 mol%. Annotation with Prokka revealed the presence of 3575 coding sequences, 3 rRNA and 50 tRNA entries (**Table 2**). In **Figure 6** the results of gene content analysis based on reciprocal blast are shown in a circular plot. Known genomes of subdivision 4 Verrucomicrobia are compared to the IG16b^T^ chromosome, thereby revealing its unique genomic regions (**Figure 6**, gray boxes). Some of these regions were also predicted to be genomic islands (**Figure 6**, gray zones, outer rim), originating from horizontal gene transfer, and mainly hold hypothetical proteins or proteins with domains of unknown function. All predicted prophage regions (**Figure 6**, yellow zones, outer rim) were incomplete (**Supplementary Table S4**), thus no intact prophage exists in the chromosome of strain IG16b^T^.

**Table 2:** Basic genome information of the newly sequenced strain IG16b^T^ and other described subdivision 4 Verrucomicrobia.

Feature	IG16b^T^	*Opitutus terrae* PB90-1^T^	*Coraliomargarita akajimensis* 04OKA010-24^T^
Size [bases]	4,199,284	5,957,605	3,750,771
Genes	3575	4612	3120
GC content [mol%]	66.5	65.3	53.6
rRNA entries	3	4	6
tRNA entries	50	65	46


### Peptidoglycan in the Verrucomicrobial Subdivision 4

#### Bioinformatic Analysis of Peptidoglycan Synthesis Genes and β-lactamase Homologs

Using comparative genomics, we analyzed the genomes of strain IG16b^T^, *O. terrae* PB90-1^T^ and *C. akajimensis* 04OKA010-24^T^ (compare **Table 2**) with respect to genes required for the synthesis of peptidoglycan (PG). Results of our blast-based approach led to the conclusion that all investigated organisms harbor almost all genes essential for the synthesis of PG (**Supplementary Table S5**). Interestingly, for the penicillin binding proteins only ftsI was identified above threshold. Gene products of *murB* and *murC* were encoded polycistronic in IG16b^T^, *O. terrae* and *C. akajimensis* (compare **Supplementary Table S5**, orange boxes) leading to the identification of the same protein when investigated with the query protein sequences for MurB and MurC.

Tolerance of β-lactam-derived antibiotic agents in bacteria is often related to one of several modes of resistance, including efflux or exclusion mechanisms, alterations in target proteins or the most common cause being the presence of β-lactamases to degrade the antibiotic compound (Poole, 2004). Growth of strains IG15^T^, IG16b^T^, and IG31^T^ on solid media supplemented with the β-lactam carbenicillin gave rise to the assumptions that these strains possess a mode of tolerance against β-lactams. Employing comparative genomics, we analyzed the presence of β-lactamase genes in the genomes of strain IG16b^T^, *O. terrae* and *C. akajimensis* (see **Supplementary Tables S6** and **S7**). For IG16b^T^ and *O. terrae*, three β-lactamases were identified, while for *C. akajimensis* no β-lactamase was found with the tested criteria. Our findings suggest that a tolerance mechanism against carbenicillin exists in strain IG16b^T^ and is at least partially due to the presence of β-lactamases, leading to the survival of the organism until the antibiotic agent is decayed from the cultivation medium.

#### Lysozyme Susceptibility Assay

**FIGURE 7:**
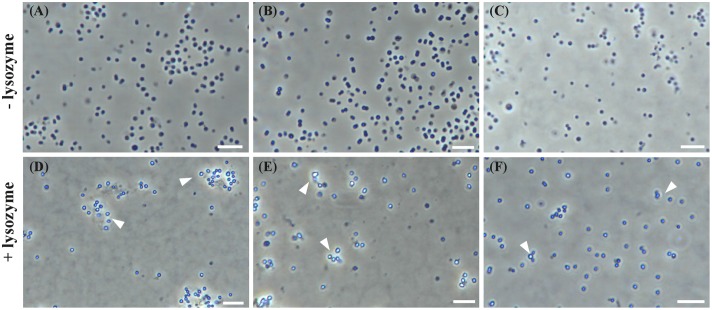
**Effect of lysozyme treatment on strains IG15^T^, IG16b^T^, and IG31^T^.** Cells of all three strains were incubated with lysozyme. Untreated cells served as negative controls. Cell morphology was investigated by light microscopy. All strains were susceptible to lysozyme treatment, leading to disruption of the cells shape (**D–F;** white arrowheads), while cells in negative controls remained healthy during the whole incubation time **(A–C)**. The isolates showed different resistance to the lysozyme treatment. Cells of isolate IG15^T^ **(D)** and IG31^T^ **(F)** were disrupted after 24 h of incubation in M1H medium or ddH_2_O, respectively. In contrast, cells of strain IG16b^T^ **(E)** lysed after 3 h of incubation in M1H medium. Scale bar indicates 5 μm.

Treatment with lysozyme leads to the disruption of the cell envelope by hydrolytic cleavage of β-1,4-linkages in the peptidoglycan complex (Johnson et al., 1968). Untreated cells of strains IG15^T^, IG16b^T^, and IG31^T^ maintained typical coccoid cell morphology, while all three strains displayed a loss of mobility during incubation at 37°C (**Figures 7A–C**, respectively). Cells that were treated with lysozyme for up to 24 h at 37°C in either culture medium (IG15^T^ and IG16b^T^) or ddH_2_O (IG31^T^) showed different susceptibility levels toward the lysozyme treatment. Cells of strain IG15^T^ showed no lysis in M1H medium after 1, 3, or 6 h, but were lysed after 24 h of incubation (**Figure 7D**; white arrowheads). Cells of strain IG16b^T^ were destroyed after 3 h incubation in M1H medium (**Figure 7E**; white arrowheads). Since strain IG31^T^ showed no lysis after 24 h in M1H medium, osmotic stress was increased by incubation of cells in ddH_2_O and cells were disrupted in ddH_2_O after 24 h (**Figures 7D,F**; white arrowheads).

#### Biochemical Evidence for the Presence of Peptidoglycan Building Blocks

**FIGURE 8:**
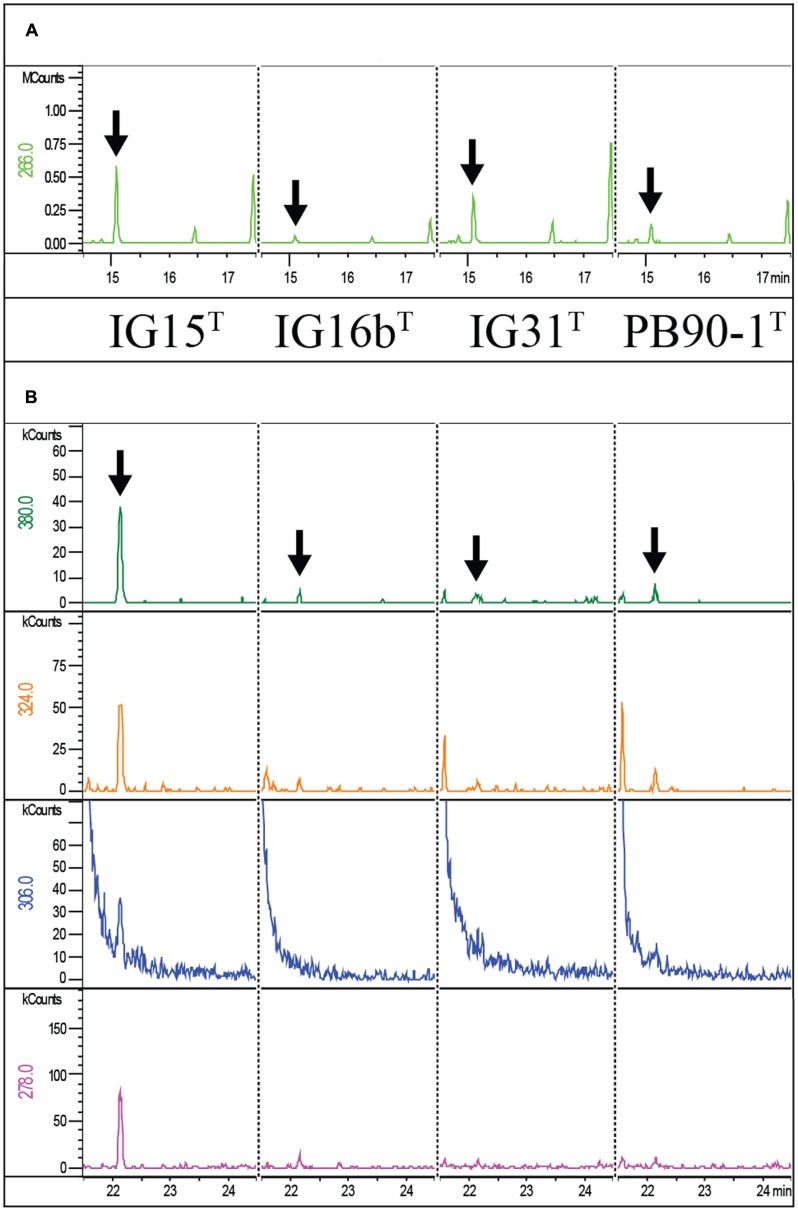
**Mass spectrometric detection of diaminopimelic acid and ornithine in IG15^T^, IG16b^T^, IG31^T^ and *O. terrae* PB90-1^T^.** Extracted Ion chromatograms of ornithine **(A)** and the DAP derivative (*N*-heptafluorobutyryl DAP isobutylester) **(B)** from whole-cell hydrolysates of strains IG15^T^, IG16b^T^, IG31^T^ and *O. terrae* PB90-1^T^ are shown. Masses of the ornithine fragment (266 m/z) were detected for IG15^T^, IG16b^T^, IG31^T^, *O. terrae* PB90-1^T^ at 15.13 min retention time. Masses of DAP fragments (380, 324, 306, and 278 m/z) were detected for IG15^T^, IG16b^T^, IG31^T^ and *O. terrae* PB90-1^T^ at 22.17 min retention time. Peaks confirming the presence of amino acids are highlighted for ornithine (**A**; black arrows) and DAP (**B**; black arrows) for all strains analyzed.

First, the presence of DAP was investigated for strains IG15^T^, IG16b^T^, and IG31^T^ by TLC and no DL-DAP was detected. In contrast, Gram-negative and Gram-positive reference strains, *E. coli* DSM498 and *B. subtilis* DSM10, respectively, showed signals for DAP (**Supplementary Figure S3**), with *E. coli* giving only a weak signal. However, we analyzed whole-cell hydrolysates of IG15^T^, IG16b^T^, and IG31^T^ using a more sensitive GC/MS method that previously revealed DAP in Planctomycetes. Despite negative results in TLC, we found the specific ion peaks, characteristic for DAP (compare **Figure 8B**), indicating the presence of peptidoglycan in IG15^T^, IG16b^T^, and IG31^T^ and *O. terrae* PB90-1^T^. The same ion peaks were previously detected for *E. coli* DSM 498 (Spring et al., 2016), the identical *E. coli* strain we here used in our TLC experiment. In addition, ornithine was detected in the whole cell hydrolysates of all three novel strains and the closest related type strain, *O. terrae* (**Figure 8A**). A quantitative estimation, based on the internal standard used, revealed that DAP and ornithine occurred in nearly equivalent, albeit low amounts in strains IG15^T^ and IG16b^T^ while ornithine was the dominant substance detected for *O. terrae* and strain IG31^T^ (**Table 3**). However, quantities of DAP for strains IG15^T^ (7 nmol), IG16b^T^ (6 nmol), IG31^T^ (3 nmol) and *O. terrae* (4 nmol) were nearly 10-fold lower than those detected for the control *E. coli* strain (63 nmol), investigated in the study of Spring et al. (2016), which explains why no signal of DAP was visible in TLC experiments for strains IG15^T^, IG16b^T^, and IG31^T^, but a weak signal for *E. coli* (compare **Supplementary Figure S3**). Furthermore, proteins essential for DAP biosynthesis via the aminotransferase pathway are present in the genome of strain IG16b^T^ (LysC :WP_069962807.1, WP_069963418.1; Asd: WP_069963129.1; DapA: WP_069962952.1; DapB: WP_069962953.1; DapL: WP_069960938.1; DapF: WP_069963382.1) as well as a alanine racemase (WP_069962553.1).

**Table 3:** Content of diagnostic diamino acids of peptidoglycan in whole-cell hydrolysates of *Opitutus terrae* PB90-1^T^ and strains IG15^T^, IG16b^T^, and IG31^T^.

Organism	DAP	Orn
*Opitutus terrae* PB90-1^T^	4	11
IG15^T^	7	9
IG16b^T^	6	6
IG31^T^	3	18


*Values are nmol of diamino acid per 1.0 mg lyophilized biomass. DAP, diaminopimelic acid; Orn, ornithine.*

Thus, we conclude despite negative results in TLC, that all analyzed strains contain DAP as diagnostic diamino acid of peptidoglycan. Additionally, ornithine was detected which is a part of the peptidoglycan backbone of certain gram-negative bacteria (Yanagihara et al., 1984; Spring et al., 2016).

#### Cell Sacculi of IG16b^T^

**FIGURE 9:**
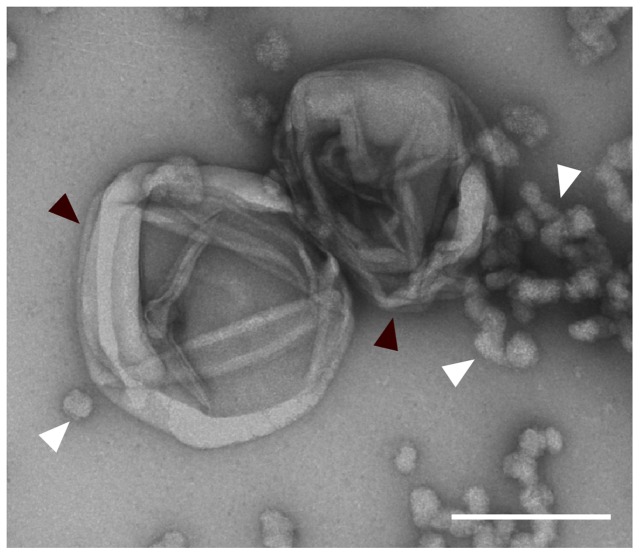
**Extracted peptidoglycan sacculus of strain IG16b^T^.** Cells were boiled in 4% SDS for 1 h and unbound SDS was dialyzed against ddH_2_O over the course of 3 days. Sacculi were negatively stained with 1% aqueous uranyl acetate and imaged by transmission electron microscopy (black arrowheads). Protein-bound SDS is seen in the sample (white arrowheads). Scale bar indicates 0.2 μm.

To give the ultimate proof that PG exists in the novel strains isolated in this study cell sacculi were extracted from strain IG16b^T^ and investigated by TEM. TEM imaging revealed the presence of cell sacculi (**Figure 9**; **Supplementary Figure S4**) with remaining protein accumulations (white arrowheads) being present in the sample investigated.

The isolation of PG sacculi together with the presence of DAP and PG synthesis genes, suggests that the claim of verrucomicrobial subdivision 4 lacking peptidoglycan, is not entirely justifiable (compare **Table 4**).

**Table 4:** Taxonomic affiliation and detected presence of peptidoglycan (DAP) in strains IG15^T^, IG16b^T^, IG31^T^ and validly published type strains within subdivision 4 Verrucomicrobia.

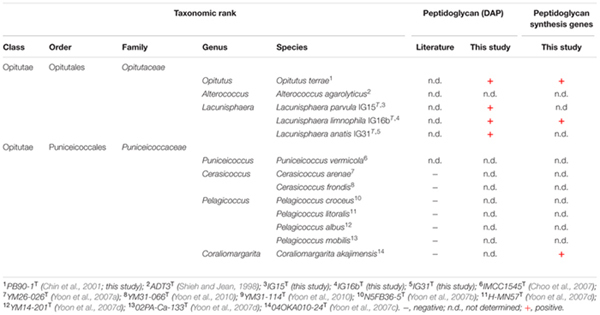

## Discussion

All free-living bacteria possess a peptidoglycan cell wall (PG) to withstand environmental osmotic challenges and to maintain cell shape (Vollmer et al., 2008), with subdivision 4 Verrucomicrobia being described as one of the few exceptions (Yoon, 2011). Since it was recently demonstrated that Planctomycetes possess a PG cell wall (Jeske et al., 2015) despite oppositional previous reports (König et al., 1984), we revisited the question if subdivision 4 Verrucomicrobia are indeed an exception to this otherwise universal cell biological bacterial trait. Given that only a few representatives of the verrucomicrobial subdivision 4 are available in axenic culture, we applied a selective β-lactam-based cultivation approach considering the putative lack of PG to specifically enrich subdivision 4 Verrucomicrobia from a limnic water sample. As β-lactam antibiotics prevent PG formation and remodeling during cell division by irreversible interaction with penicillin-binding proteins involved in the final step of PG synthesis (Waxman and Strominger, 1983), subdivision 4 Verrucomicrobia should comprise intrinsic resistance if no PG *per se* exists. Accordingly, all three strains described in this study were obtained from plates initially containing carbenicillin. However, all novel verrucomicrobial strains grew only after 4 months of incubation, indicating rather antibiotic degradation through hydrolysis than an intrinsic resistance against β-lactam antibiotics. Thus, we analyzed the genome of the novel strain IG16b^T^ in more detail to reveal the nature of its cell wall architecture and possible resistance mechanism to β-lactam antibiotics. First, we employed bioinformatics and found strain IG16b^T^ to encode β-lactamase proteins that can confer resistance against β-lactam antibiotics such as carbenicillin. Second, we incubated cultures of strains IG16b^T^ and IG15^T^ with carbenicillin concentrations of 500–2000 mg/l, which were far above the 100 mg/l working concentration usually used as selection pressure for β-lactamase mediated resistance in molecular laboratory approaches (Green and Sambrook, 2012). Accordingly, SEM analysis revealed that carbenicillin treated cells were inhibited in cell division and increased in size. However, they withstood the antibiotic reagent and resumed growth, once carbenicillin was depleted from the cultivation medium, as happened through hydrolysis over time once they were initially isolated from the environment. Similar behavior has been observed for *Chlamydia psittaci*, where presence of penicillin led to swelling of reticulate bodies and incomplete cell division, while cells transferred to penicillin-free medium resumed division (Matsumoto and Manire, 1970). Our observations thus rather suggest a mode of tolerance, possibly enabled by β-lactamases, than a mode of intrinsic resistance due to the absence of PG in the novel isolates. In case of intrinsic resistance, increase of carbenicillin concentration would have had no effect on cell division. However, the degradation capability of β-lactamases can be titrated to a point, where the enzyme cannot confer resistance anymore and the cell becomes affected as observed in changes of morphology in this study. Thus, this finding provided us with the ample motivation to further analyze PG in our strains. To do this comprehensively, we analyzed the genome of strain IG16b^T^ (obtained in this study) along with the published genomes of *O. terrae* (van Passel et al., 2011) and *C. akajimensis* (Mavromatis et al., 2010) employing complementary blast methods that were previously used to identify PG synthesis related genes in Planctomycetes (Jeske et al., 2015). We found that *O. terrae. C. akajimensis* and strain IG16b^T^ harbor nearly all genes essential for the synthesis of PG. Thus, from a genomic perspective based on all available type strain genomes, it is likely that subdivision 4 Verrucomicrobia can synthesize PG.

Employing a previously described procedure (Jeske et al., 2015), we next demonstrated that all three novel strains, IG15^T^, IG16b^T^, and IG31^T^, are susceptible to the treatment with lysozyme, an enzyme that destroys beta-1,4 glycosidic bonds in the peptidoglycan structure, leading to disruption of the bacterial cell envelope (Johnson et al., 1968). Our results indicate different tolerance levels of strains IG15^T^, IG16b^T^, and IG31^T^ against lysozyme, under laboratory culture conditions (in M1H medium) or under osmotic stress (IG31^T^ in ddH_2_O).

Even though all evidence so far points toward the existence of an peptidoglycan cell wall in strains IG15^T^, IG16b^T^, and IG31^T^, we obtained no signals for the diagnostic peptidoglycan-specific structural element DAP when performing TLC experiments. This result is consistent with previous reports that led to the conclusion that subdivision 4 Verrucomicrobia lack PG (Yoon et al., 2007a). However, we analyzed whole-cell hydrolysates of our strains and *O. terrae* PB90-1^T^ using a modified version –capable of quantification- of a highly sensitive method based on gas chromatography and mass spectrometry (GC/MS) detection that previously revealed DAP in Planctomycetes (Jeske et al., 2015). We found the specific ion set characteristic for DAP, while quantification of DAP in whole-cell hydrolysates of our strains and *O. terrae* revealed that this marker was only present in low quantities, possibly explaining why less sensitive methods such as TLC failed to detect DAP. Furthermore, all proteins essential for DAP synthesis were detected within the genome of strain IG16b^T^. In addition, we surprisingly detected the non-proteinogenic diamino acid ornithine that was, until recently, thought to be an exception in PG among Gram-negative bacteria limited to *Spirochaetaceae* (Schleifer and Joseph, 1973; Yanagihara et al., 1984). At this point, it cannot be excluded that ornithine could have been extracted from certain amino lipids or other cell components instead of peptidoglycan, because only whole-cell hydrolysates were analyzed. However, ornithine was recently identified in whole-cell hydrolysates of both, the proposed phylum Kiritimatiellaeota -formally known as verrucomicrobial subdivision 5- and representatives of the phylum Lentisphaerae (Spring et al., 2016), indicating that more Gram-negative bacteria display such alterations in their PG cell walls. Spring et al. additionally analyzed whole-cell hydrolysates of *E. coli* DSM 498, the same strain we used for TLC analysis, and found much higher quantities of DAP (63 nmol) then we did for our strains (compare **Table 3**), consequently supporting the observation of TLC being a method unfit to detect DAP in cases were only low quantities are present in the cell walls of the investigated organism.

To ultimately proof the existence of peptidoglycan sacculi, we isolated them from strain IG16b^T^ and visualized them employing TEM (**Figure 9**).

Based on our findings, we conclude that subdivision 4 Verrucomicrobia do possess PG sacculi. Contrary previous reports used methods such as TLC (Yoon et al., 2007c) that did not detect DAP in subdivision 4 Verrucomicrobia in our hands as well (**Supplementary Figure S3**). Thus, future analyses must meet a new standard in PG detection, set by others and us, to justify the claim that a certain free-living bacterial strain lacks PG (Pilhofer et al., 2013; Jeske et al., 2015; Packiam et al., 2015; van Teeseling et al., 2015).

Based on recent results (Pilhofer et al., 2013; Jeske et al., 2015; Packiam et al., 2015; van Teeseling et al., 2015) and the outcome of this study we further postulate -applying the *lex parsimoniae*- that all free-living bacteria require a PG cell wall to maintain cell shape integrity in habitats with osmotic conditions different from their cytosol.

### Description of *Lacunisphaera* gen. nov.

*Lacunisphaera* (La.cu.ni.sphae.ra N.L. fem. n. *lacuna*, a little lake, referring to the origin of the organism; N.L. fem. n. *sphaera*, a ball, globe, sphere; N.L. fem. n. *Lacunisphaera*, a spherical microorganism from a lake).

Cells are Gram-negative, aerobic cocci. Mono- or diplococcic are formed, but no chains or rosettes. Cells are motile during exponential growth phase, but not in late stationary phase. No spore formation was observed. Members test positive for cytochrome oxidase activity, but show no catalase activity in reaction with H_2_O_2_. Extracellular matrix formation in liquid culture is observed for some members when cultured under constant agitation. This is not true for the type species. The molar G + C content is between 65 and 67 mol%. Members contain peptidoglycan with DAP and ornithine as diamino acids. The predominant cellular fatty acid of the type species is iso-C_15:0_. Members belong to the phylum Verrucomicrobia, class *Opitutae*, order *Optitutales*, family *Opitutaceae*. The type species of the genus is *Lacunisphaera limnophila*.

### Description of *Lacunisphaera parvula* sp. nov.

*Lacunisphaera parvula* (*par.vu.la*, L. adj. *parvula* small, referring to the size of individual cells).

Main attributes are as given for the genus. Colonies grown on M1H agar were round, smooth and cream colored, while aging colonies became translucent. An extracellular matrix compound is produced in liquid cultures when kept under constant agitation, but formation was not observed on solid media. Cells are present as mono- or diplococci, but form aggregates when embedded in the extracellular matrix compound. Single cells measured 0.6 ± 0.1 μm in diameter. Substrates utilized were D-cellobiose, maltose, gentiobiose, α-D-lactose, sucrose, turanose, lactulose, succinic acid, thymidine, inosine, uridine, succinic acid mono-methyl ester, L-rhamnose, D-alanine, malonic acid, L-aspartic acid, D-melibiose, β-methyl-D-glucoside, D-fructose, α-D-glucose, DD-trehalose, bromosuccinic acid, α-cyclodextrin, dextrin, tween 80, α-ketoglutaric acid, D-gluconic acid, D-sorbitol, glycogen, D-galactose, D-raffinose, α-D-glucose-1-phosphate, D-mannose, D-glucosaminic acid, D-saccharic acid, L-alanine, D,L-lactic acid, D-galacturonic acid, D-glucuronic acid and succinamic acid. Cells grew in M1H medium at temperatures between 12 and 38°C, while 33°C was the optimum. Cells did not grow below 10°C and above 38°C. pH values between 6.0 and 9.0 were tolerated for growth, while the optimum was between 7.5 and 8.0. Major cellular fatty acids were iso-C_15:0_ (33.3%), C_16:0_ (10.2%), iso-C_13:0_ 3-OH (8.7%), C_16:1_ ω5c (8.4%) and iso-C_11:0_ (4.9%). The G + C content of the DNA of the type strain is 65.9 mol%. The type strain is IG15^T^ (=DSM 26814 = LMG 29468) and was isolated from the surface water column of a freshwater lake during a cyanobacterial blooming event.

### Description of *Lacunisphaera limnophila* sp. nov.

*Lacunisphaera limnophila* (*lim.no’ phi.la* Gr. n. *limnos* lake*;* Gr. adj. *philus* loving*;* N.L. adj. *limnophila* lake loving).

Overall characteristics are as described for the genus. Colonies grown on M1H agar were round, smooth and cream colored, while aging colonies became translucent. Liquid cultures appeared pale yellowish. Cells are present as mono- or diplococci and form no chains or rosettes. Single cells measured 0.9 ± 0.2 μm in diameter. Substrates utilized were D-cellobiose, maltose, gentiobiose, α-D-lactose, sucrose, turanose, lactulose, thymidine, inosine, glycyl-L-aspartic acid, glucuronamide, pyruvic acid methyl ester, L-leucin, hydroxyl-L-proline, D-melibiose, β-methyl-D-glucoside, D-fructose, α-D-glucose, D-trehalose, glycogen, D-galactose, D-raffinose, α-D-glucose-1-phosphate and D-mannose. Cells grew in M1H medium at temperatures between 13 and 36°C, while 32°C was the optimum. Cells did not grow below 10°C and above 36°C. pH values between 6.0 and 9.0 were tolerated for growth, while the optimum was between 7.5 and 8.0. Major cellular fatty acids were iso-C_15:0_ (48.6%), Anteiso-C_15:0_ (12.1%), iso-C_15:1_ ω9c (10.3%), iso-C_13:0_ 3-OH (6.6%) and iso-C_13:0_ (5.0%). The genome based G + C content of coding sequences is 66.5 mol%. The type strain is IG16b^T^ (=DSM 26815 = LMG 29469) and was isolated from the particle-containing fraction of surface water from a freshwater lake. The 4,199,284 bp genome of *L. limnophila* IG16b^T^ was similar, yet distinct from other sequenced verrucomicrobial species in terms of gene content (**Figure 6**). In most cases, such differences were associated with genomic islands which indicate frequent horizontal gene transfer.

### Description of *Lacunisphaera anatis* sp. nov.

*Lacunisphaera anatis* (*a.na.tis* L. fem. n. *anatis* with the ducks, referring to the term ‘duck pond’ describing a lake or pond inhabited by ducks).

Overall characteristics are as described for the genus. Colonies grown on M1H agar were round, smooth and cream colored, while aging colonies became translucent. Cells are present as mono- or diplococci and form no chains or aggregates. Single cells measured 0.6 ± 0.1 μm in diameter. Substrates utilized were glycyl-L-glutamic acid, succinic acid, thymidine, inosine, uridine, succinic acid mono-methyl ester, L-rhamnose, and D-fructose. Cells grew in M1H medium at temperatures between 15 and 36°C, while 30°C was the optimum. Cells did not grow below 12°C and above 36°C. pH values between 6.0 and 9.0 were tolerated for growth, while the optimum was between 7.5 and 8.0. Major cellular fatty acids were iso-C_14:0_ (15.4%), iso-C_14:0_ (15.4%), C_16:0_ (12.1%), Anteiso-C_15:0_ (10.6%) and iso-C_16:0_ (10.1%). The G + C content of the DNA of the type strain is 67.2 mol%. The type strain is IG31^T^ (=DSM 103142 = LMG 29578) and was isolated from surface freshwater containing biomass of a cyanobacterial bloom.

## Author Contributions

PR did most of the experimental laboratory work and wrote the main part of the manuscript body and functions as first author. IG helped with the isolation of the novel strains and with cultivation measurements. CB performed all light microscopic work and cell size measurements. OJ conducted blast analysis for the detection of peptidoglycan synthesis genes and ß-lactamases. SW analyzed the gene content and generated substrate utilization heatmaps. RR coordinated sequencing of the IG16b genome sequence with Pacific Bioscience. PS did the gas chromatographic analysis of PG in the new strains. MR performed scanning electron microscopy experiments. SS cultivated reference strains for GC MS and fatty acid analysis and contributed in literature research toward ornithin in PG layers. FG analyzed sequencing data and was involved in genome assembly of IG16b’s genome sequence. CJ is PI and together with MJ functions as corresponding author. MJ and CJ, along with PR designed the study and helped with experimental setups and design.

## Conflict of Interest Statement

The authors declare that the research was conducted in the absence of any commercial or financial relationships that could be construed as a potential conflict of interest.
