# Supplementary material for: Three Novel Species with Peptidoglycan Cell Walls form the New Genus Lacunisphaera gen. nov. in the Family Opitutaceae of the Verrucomicrobial Subdivision 4
Source: Front Microbiol. 2017 Feb 13;8:202. doi: 10.3389/fmicb.2017.00202 (PMC5303756; doi:10.3389/fmicb.2017.00202)
Supplement: Supplementary file 5 [file Data_Sheet_1.PDF]

Figure S1 | Temperature optima of IG15^T, IG16b^T and IG31^T. To determine the optimum growth temperature, optical density was measured at 600 nm (OD_{600nm}). Slope values, corresponding to change of OD_{600nm} during exponential growth phase, were plotted against the corresponding temperature value. Temperature optima of IG15^T (**A**), IG16b^T (**B**) and IG31^T (**C**) were determined to be 33°C, 32°C and 30°C respectively. Each dot represents the mean of triplicate measurements.

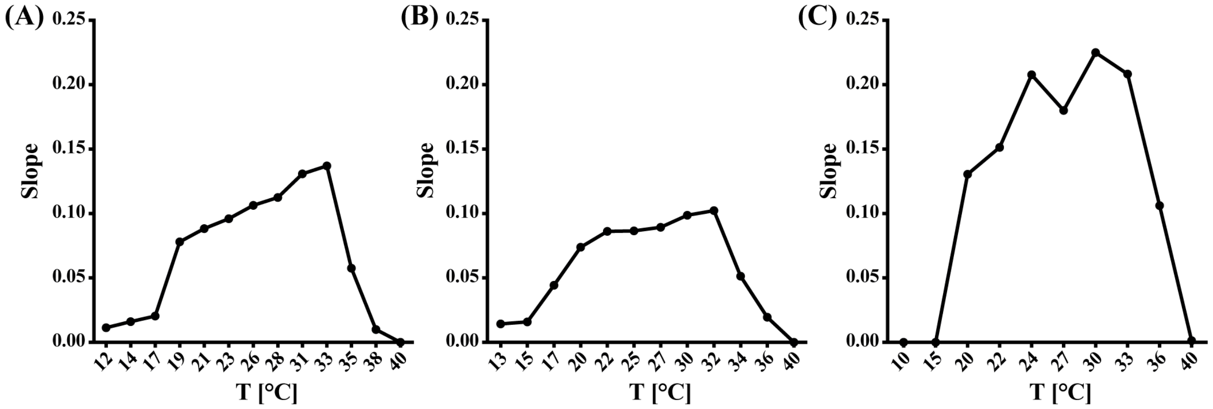

Figure S2 | Growth curves and cell counts of IG15^T and IG16b^T in the presence of carbenicillin. IG15^T (A) and IG16b^T (B) cultures were inoculated with 500 (grey squares), 1000 (grey triangle) or 2000 (tilted triangle) mg/l carbenicillin, while the positive controls (black dots) remained untreated. Points of measurement represent the mean of triplicates. While optical density curves indicate bacterial growth, albeit reduced in treated samples, at all concentrations of the antibiotic agent, cell counts of end point measurements (C) show a significant difference (p<0.001) between control and 2000 mg/l cultures (~10-fold higher cell counts for untreated samples).

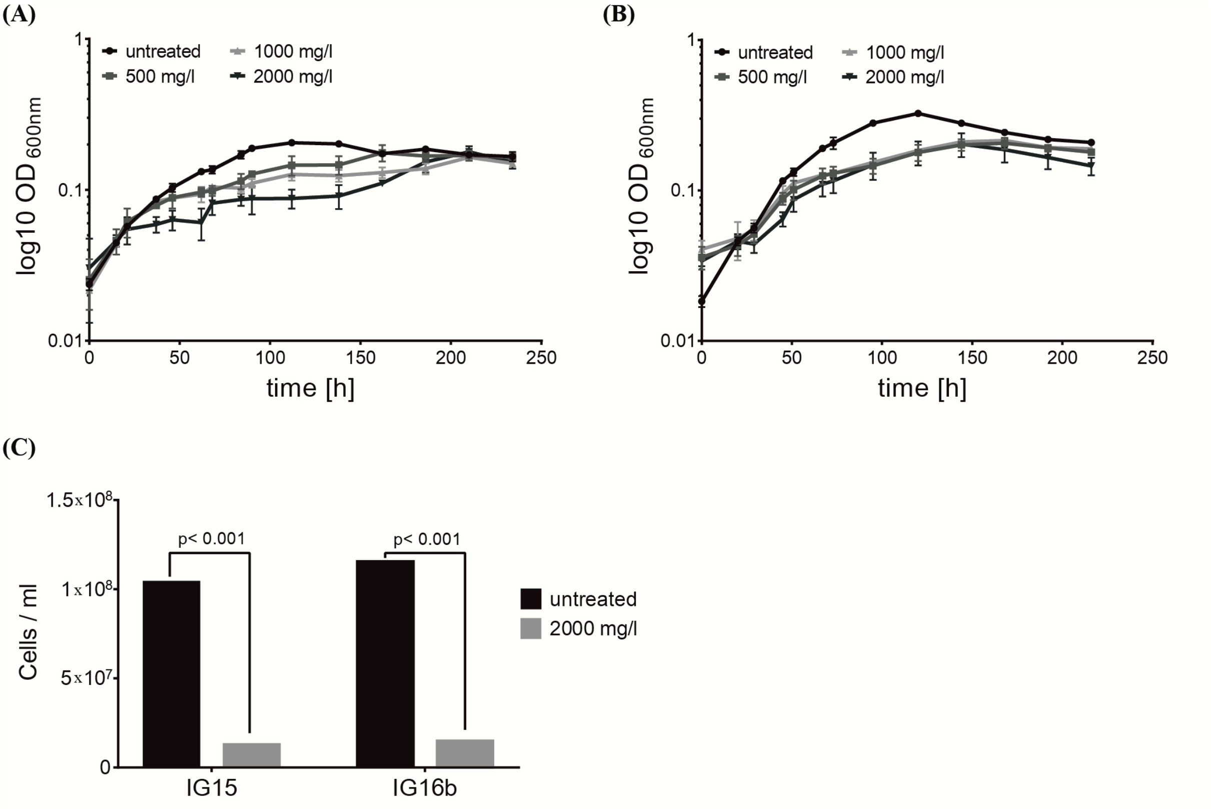

Figure S3 | Thin layer chromatogram of IG15^T, IG16b^T and IG31^T. Thin layer chromatography was used to detect diaminopimelic acid (DAP) from whole cell hydrolysates of strains IG15^T, IG16b^T and IG31^T. Cell hydrolysates of IG15^T, IG16b^T and IG31^T are devoid of DAP and show no bands corresponding to stereoisomers of DAP, indicating the absence in all three strains. The chromatogram of *Bacillus subtilis* DSM10 shows a distinct band for DAP while *Escherichia coli* DSM498 shows only a weak signal in comparison to the substance standard used (Std.). The dotted line between the chromatogram of strain DSM 498 and the other strains investigated indicates that analyses were performed using the same experimental setup, but were run on different cassettes and resulting images were combined afterwards using the software Adobe Illustrator CC (Adobe Systems Software, Dublin, Ireland).

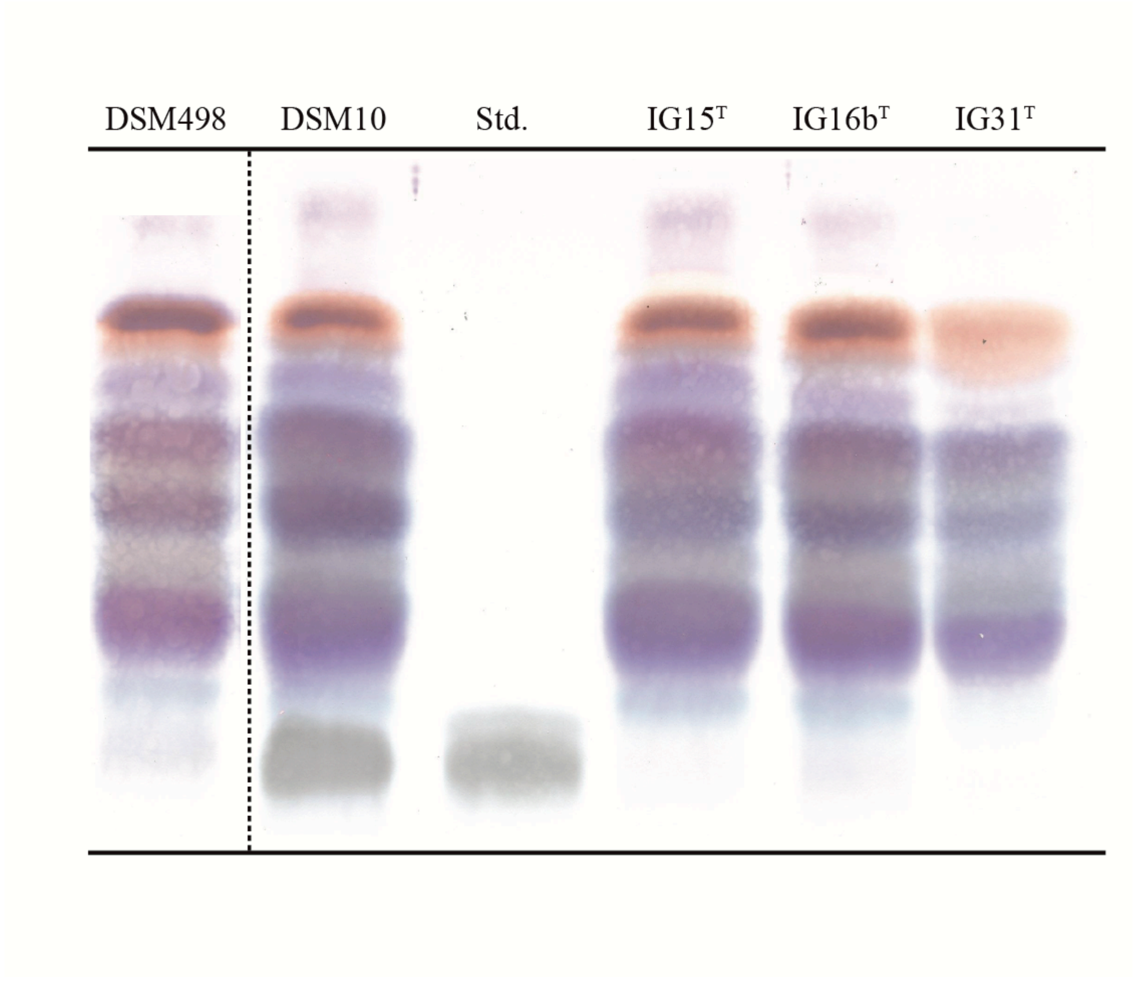

Figure S4 | Morphology of IG16b^T cell sacculi. Cell sacculi were prepared by boiling cells in 4% SDS for 1 hour. Unbound SDS was removed by dialysis for 3 days against ddH_2O. Sacculi were negatively stained with 1% aqueous uranyl acetate and imaged by transmission electron microscopy (A-F). Protein-bound SDS can be seen as clumps. Scale bar indicates 0.2 μm.

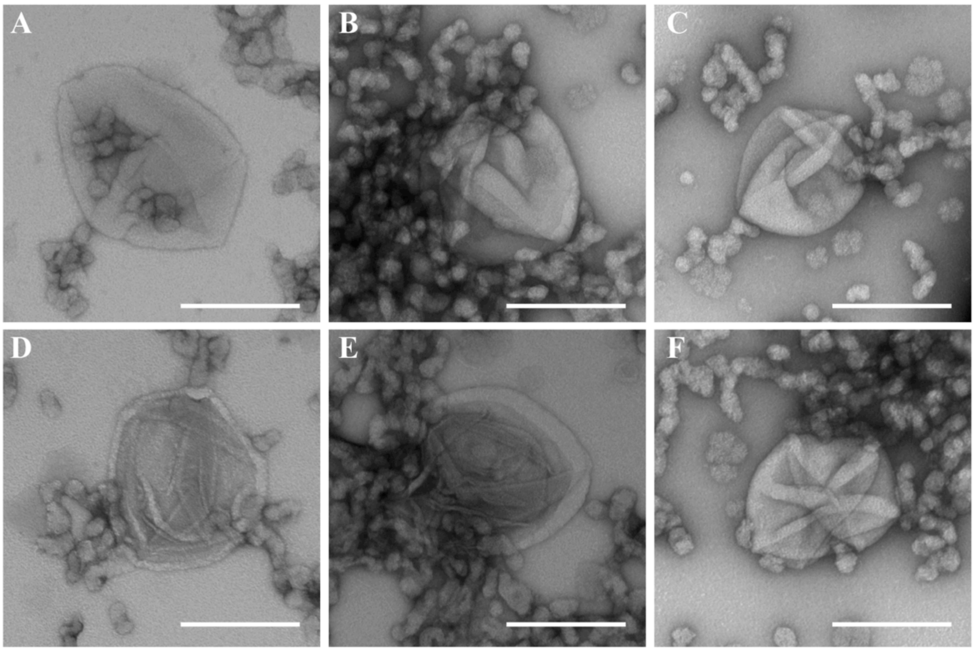

TABLE S1 | Additional primers used to generate near full-length sequences of verrucomicrobial isolates. Sequences were assembled using the ContigExpress application of the Vector NTI® Advance 10 software.

**Primer designation**	**Sequence** (5'→ 3')	**Reference**
341f	CCT ACG GGW GGC WGC AG	modified from Muyzer *et al.*, 1993
515f	GTG CCA GCA GCC GCG G	modified from Lane, 1991
515r	CCG CGG CTG CTG GCA C	modified from Muyzer *et al.*, 1993
1055f	ATG GCT GTC GTC AGC T	modified from Lee, 1993
1055r	AGC TGA CGA CAG CCA T	modified from Lee, 1993

References:

Lane, D.J. (1991) 16S/23S rRNA sequencing. In: Stackebrandt, E., Goodfellow, M., Nucleic acid techniques in bacterial systematic *Wiley*. 115-175

Lee, S., Malone, C., Kemp, P.F. (1993) Use of multiple 16s rRNA-targeted fluorescent probes to increase signal strength and measure cellular RNA from natural planktonic bacteria *Mar. Ecol. Prog. Ser.* 101: 193-201

Muyzer, G., de Waal, E.C., Uitterlinden, A. G. (1993): Profiling of complex microbial populations by denaturing gradient gel electrophoresis analysis of polymerase chain reaction-amplified genes coding for 16S rRNA *Appl. Environm. Microbiol.* **59(3):** 695

Table S2 | List of reference strains used for 16S ribosomal RNA gene phylogenetic tree reconstruction. Strain designations and accession numbers were obtained from the NCBI database and the List of Procaryotic names with Standing in Nomenclature website (http://www.bacterio.net/-index.html).

Species	Strain	16S rRNA gene accession number
Akkermansia muciniphila	Muc^T	AY271254
Alterococcus agarolyticus	ADT3^T	AF075271
Brevifollis gellanilyticus	DC2c-G4^T	NR113149
Cerasicoccus arenae	YM26-026^T	AB292183
Cerasicoccus frondis	YM31-066^T	AB372850
Cerasicoccus maritimus	YM31-114^T	AB372849
Chthoniobacter flavus	Ellin428^T	NR115225
Coraliomargarita akajimensis	04OKA010-24^T	CP001998
Haloferula chungangensis	CAU 1074^T	JN001489
Haloferula harenae	YM23-227^T	AB372852
Haloferula helveola	05IJR53-1^T	AB372855
Haloferula luteola	YC6886^T	FJ032193
Haloferula phyci	AK18-024^T	AB372854
Haloferula rosea	06SJR1-1^T	AB372853
Haloferula sargassicola	MN1-1037^T	AB372856
Limisphaera ngatamarikiensis	NGM72.4^T	HF947551
Luteolibacter algae	A5J-41-2^T	AB331893
Luteolibacter arcticus	MC 3726^T	KP101281
Luteolibacter cuticulihirudinis	E100^T	JQ429496
Luteolibacter luojiensis	DR4-30^T	JN630810
Luteolibacter pohnpeiensis	A4T-83^T	AB331895
Luteolibacter yonseiensis	EBTL01^T	JQ319003
Methylacidimicrobium cyclopophantes	3B^T	NR126315
Methylacidimicrobium fagopyrum	3C^T	NR126313
Methylacidimicrobium tartarophylax	4AC^T	NR126314
Methylacidiphilum fumariolicum	SolV^T	EF591088
Methylacidiphilum infernorum	V4^T	NR074583
Methylacidiphilum kamchatkense	Kam1^T	EF127896
Opitutus terrae	PB90-1^T	AJ229235
Pedosphaera parvula	Ellin514^T	AY960777
Pelagicoccus albus	YM14-201^T	AB286016
Pelagicoccus croceus	N5FB36-5^T	AB297922
Pelagicoccus litoralis	H-MN57^T	AB286017
Pelagicoccus mobilis	02PA-Ca-133^T	AB286015
Persicirhabdus sediminis	YM20-087^T	AB331886
Prosthecobacter algae	EBTL04^T	JQ319004
Prosthecobacter debontii	FC3^T	U60014
Prosthecobacter dejongeii	FC1^T	U60012
Prosthecobacter fluviatilis	HAQ-1^T	AB305640
Prosthecobacter fusiformis	FC4^T	U60015
Prosthecobacter vanneervenii	FC2^T	U60013
Puniceicoccus vermicola	IMCC1545^T	DQ539046
Roseibacillus ishigakijimensis	MN1-741^T	AB331888
Roseibacillus persicicus	YM26-010^T	AB331890
Roseibacillus ponti	YM27-120^T	AB331889
Roseimicrobium gellanilyticum	DC2a-G7^T	AB552861
Rubritalea halochordaticola	MN1-1006^T	AB543683
Rubritalea marina	Pol012^T	DQ302104
Rubritalea sabuli	YM29-052^T	AB353310
Rubritalea spongiae	YM21-132^T	AB297805
Rubritalea squalenifaciens	HOact23^T	AB277853
Rubritalea tangerina	YM27-005^T	AB297806
Terrimicrobium sacchariphilum	NM-5^T	NR133878
Verrucomicrobium spinosum	IFAM 1439^T	X90515
Chlamydia trachomatis	A/Har-13^T	D89067
Parachlamydia acanthamoebae	Bn9^T	Y07556
Phycisphaera mikurensis	FYK2301M01^T	NC017080
Simkania negevensis	Z^T	U68460
Victivallis vadensis	Cello^T	AY049713
Waddlia chondrophila	WSU 86-1044^T	AF042496
Bacterium RS12A	RS12A	AB360430
Bacterium RS58G	RS58G	AB360416
Bacterium RS5A	RS5A	AB360425
Uncultured bacterium clone (water)	147ds20	AY212598
Uncultured bacterium clone (wetland)	SEAB1AB091	KC432279
Uncultured bacterium clone (root)	SRRB12	AB240503
Uncultured bacterium clone (seep)	IS-77	GQ339177
Uncultured bacterium clone (sludge)	0131	AB286360

TABLE S3 | Cellular fatty acid contents (%) of strain IG15T^{T}, IG16bT^{T} and IG31T^{T} investigated in this study.

Fatty acid	1	2	3
**Saturated**			
$C_{14:0}$	1.72	0.17	1.17
$C_{15:0}$	-	-	-
$C_{16:0}$	10.18	1.13	12.09
$C_{17:0}$	0.51	0.10	0.76
$C_{18:0}$	0.46	-	0.21
**Unsaturated**			
$C_{15:1\ \omega 6c}$	-	-	-
$C_{16:1\ \omega 5c}$	8.35	0.35	5.42
$C_{16:1\ \omega 7c}$	0.20	0.25	3.59
$C_{16:1\ \omega 9c}$	-	1.96	-
$C_{16:1\ \omega 11c}$	3.33	-	1.49
$C_{17:1\ \omega 10c}$	-	2.10	-
$C_{18:1\ \omega 9c}$	0.75	0.14	0.15
**Branched**			
Iso-$C_{11:0}$	4.90	3.99	0.04
Iso-$C_{12:0}$	-	-	4.93
Iso-$C_{13:0}$	0.68	5.03	5.12
Iso-$C_{14:0}$	0.54	0.22	15.36
Iso-$C_{15:0}$	33.33	48.59	9.06
Iso-$C_{15:1\ \omega 9c}$	2.55	10.25	0.99
Iso-$C_{16:0}$	0.45	0.49	10.07
Iso-$C_{17:0}$	2.88	2.91	0.57
Iso-$C_{17:1\ \omega 10c}$	2.38	2.10	0.17
Anteiso-$C_{13:0}$	0.12	0.35	4.47
Anteiso-$C_{15:0}$	-	12.09	10.61
**3-Hydroxy**			
$C_{12:0}$ 3-OH	2.00	0.29	2.94
$C_{14:0}$ 3-OH	-	-	1.69
Iso-$C_{13:0}$ 3-OH	8.71	6.57	1.38
Iso-$C_{15:0}$ 3-OH	1.86	0.20	2.36

Taxa | 1, IG15T^{T} (this study); 2, IG16bT^{T} (this study); 3, IG31T^{T} (this study). Values below <1% not shown.

Tables S4-S7: Separate as Excel files
